# Good guy or bad guy: the opposing roles of microRNA 125b in cancer

**DOI:** 10.1186/1478-811X-12-30

**Published:** 2014-04-28

**Authors:** Julia Banzhaf-Strathmann, Dieter Edbauer

**Affiliations:** 1 German Center for Neurodegenerative Diseases, Site Munich, Schillerstr. 44, 80336 Munich, Germany; 2 Adolf Butenandt Institute, Biochemistry, Ludwig-Maximilians University Munich, Schillerstr. 44, 80336 Munich, Germany; 3 Munich Cluster of Systems Neurology (SyNergy), Munich, Germany

**Keywords:** MicroRNA, miR-125b, Cancer, Oncogene, Tumor suppressor gene

## Abstract

MicroRNAs (miRNAs) are a class of non-coding RNAs that post-transcriptionally silence target mRNAs. Dysregulation of miRNAs is a frequent event in several diseases, including cancer. One miRNA that has gained special interest in the field of cancer research is miRNA-125b (miR-125b). MiR-125b is a ubiquitously expressed miRNA that is aberrantly expressed in a great variety of tumors. In some tumor types, e.g. colon cancer and hematopoietic tumors, miR-125b is upregulated and displays oncogenic potential, as it induces cell growth and proliferation, while blocking the apoptotic machinery. In contrast, in other tumor entities, e.g. mammary tumors and hepatocellular carcinoma, miR-125b is heavily downregulated. This downregulation is accompanied by de-repression of cellular proliferation and anti-apoptotic programs, contributing to malignant transformation. The reasons for these opposing roles are poorly understood. We summarize the current knowledge of miR-125b and its relevant targets in different tumor types and offer several hypotheses for the opposing roles of miR-125b: miR-125b targets multiple mRNAs, which have diverse functions in individual tissues. These target mRNAs are tissue and tumor specifically expressed, suggesting that misregulation by miR-125b depends on the levels of target gene expression. Moreover, we provide several examples that miR-125b upregulation dictates oncogenic characteristics, while downregulation of miR-125b corresponds to the loss of tumor suppressive functions. Thus, in different tumor entities increased or decreased miR-125b expression may contribute to carcinogenesis.

## Introduction

The discovery of microRNAs (miRNAs) twenty years ago [1] revealed a new layer of post-transcriptional regulation of gene expression in all areas of cell biology. MiRNAs play a crucial role in epigenetic gene regulation and have been linked to development and differentiation, cell growth and cell death [2-5]. They are misregulated in several disorders, including neurodegenerative diseases, cardio-vascular diseases and cancer [2-5]. MiRNAs are small, non-coding single stranded RNAs, 21-25 nucleotides in length that are generated from a primary miRNA transcript (pri-miRNA). Often several miRNAs are clustered on one pri-miRNA transcript [6]. A nuclear ribonuclease termed Drosha excises 70-100 nucleotide hairpins, termed precursor miRNAs (pre-miRNAs). Upon nuclear export, a second ribonuclease known as Dicer cleaves the pre-miRNAs, giving rise to a miRNA duplex. Upon binding to the RNA-Induced Silencing Complex (RISC) the passenger strand is cleaved and released. The mature miRNA associated with RISC can bind to complementary mRNAs, leading to either translational repression or mRNA degradation, depending on the level of complementarity between the miRNA and the target sequence. Typically, one miRNA has multiple target mRNAs and, thus, can regulate several genes or even pathways in parallel (reviewed in [7]).

The development of novel techniques to overexpress miRNAs on the one hand, and to sequester endogenous miRNAs via “sponges” [8], “tough decoys” [9] or “antagomirs” [10] on the other hand, led to the understanding of fundamental roles of miRNAs in physiological processes as well as disease mechanisms, including cancer. Systematic miRNA profiling of human cancer samples and corresponding normal tissue, led to the identification of multiple aberrantly deregulated miRNAs in cancer. So-called miRNA signatures of miRNA expression profiles have been identified and proved to be a very useful diagnostic tool that allows for predictions of clinical outcome (reviewed in [11]).

### MiRNA 125b in cancer

One miRNA that has gained special interest in the field of cancer research is miRNA 125b (miR-125b), which is misregulated in a broad variety of tumors [12]. However, while miR-125b upregulation in some tumor entities suggests oncogenic potential of miR-125b, downregulation in other tumor types suggests that miR-125b is rather tumor suppressive.

MiR-125b is the human orthologue of *lin-4*, one of the very first miRNAs identified in *C. elegans*[1,13]. Studying *lin-4* in *C. elegans* revealed the fundamental mechanisms of miRNA signaling and regulation. *Lin-4* is essential for post-embryonic proliferation and differentiation in the worm (for further reading see excellent reviews [14,15]).

Human miR-125b is a ubiquitously expressed miRNA with highest expression levels in brain and ovaries, followed by the thyroid gland, pituitary gland, epididymis, spleen, testes, prostate, uterus, placenta and liver (see http://www.microRNA.org). MiR-125b belongs to the miR-125 family, consisting of miR-125a, miR-125b-1 and miR-125b-2 which result in almost identical products of distinct genes. Although mature miR-125a and miR-125b have different sequences, they share the same seed region (nucleotides 1-9), suggesting that they regulate the same target mRNAs. MiR-125a has been shown to be downregulated in colorectal cancer, breast cancer, gastric cancer, non-small cell lung cancer (NSCLC) and glioblastomas, the causes and consequences of which are discussed elsewhere [12].

Mature miR-125b is generated from two genes, miR-125b-1 (on chromosome 11q24) and miR-125b-2 (on chromosome 21q21). It has been demonstrated that both loci are within so-called fragile sites which are commonly deleted in breast, lung, ovary and cervical cancer [16], implying a miR-125b loss of function in those tumor types. Many other miRNA profiling studies revealed that miR-125b is also downregulated in head and neck tumors [17], gliomas [18], melanomas [19], endometrial tumors [20], oral squamous cell carcinomas [21], osteosarcomas and Ewing sarcomas [22]. However, the molecular mechanisms that lead to miR-125b downregulation in these cancer types are not fully understood. In some cases hypermethylation in the promoter regions of miR-125b has been shown to block miR-125b expression levels in cancer [23,24].

Several other tumor entities, however, show enhanced miR-125b signaling. In colorectal cancer, miR-125b upregulation is associated with poor prognosis. Furthermore, miR-125b has been shown to be upregulated in certain leukemias [25-27], gastric and follicular cancers [28,29], pancreatic cancer [30] and some brain tumor derived glioma cell lines [31,32]. The reasons for a dramatic upregulation of miR-125b in these tumor types are not very well understood. Copy number variations and chromosomal translocations occur frequently at miRNA loci leading to aberrant miRNA expression [33]. In myelodysplastic syndrome, which may progress to acute myeloid leukemia, a t (2;11) (p21; q23) translocation leads to a 6- to 90-fold upregulation of miR-125b-1 and -2 without affecting the expression of other genes located close to that region [34]. Further, in B-cell acute lymphoblastic leukemia the insertion of miR-125b-1 into a immunoglobulin heavy chain locus has been identified in one patient [35]. For other tumor types the causes for miR-125b upregulation are unknown so far.

Numerous studies have proven a role for miR-125b in proliferation, apoptosis and cellular differentiation [36,37], already suggesting a potentially important role during carcinogenesis. Recent studies demonstrated that miR-125b directly targets the tumor suppressor gene p53 [32,38], which is essential to maintain genome stability and plays a central role in regulation of apoptosis (reviewed in [39]). This direct interaction has been demonstrated for human and zebrafish p53, however there is no conserved miR-125b seed region in the mouse p53 3’UTR. Yet, it has been demonstrated that miR-125b interacts with multiple mRNAs, including apoptosis regulators, such as Bak1 and Puma as well as cell cycle reglators, such as cyclin C and cdc25c that belong to the p53 network in human, zebrafish and mouse [37]. Thus, by inhibiting key players of the p53 network across several species, miR-125b interferes with multiple cancer relevant pathways.

Other oncogenic signaling molecules that are relevant for tumorigenesis have been described to be directly targeted by miR-125b. The verified target genes v-erb-b 2 and 3 avian erythroblastic leukemia viral oncogene homolog (ERBB2/3), also named human epidermal growth factor receptor (Her) 2 and 3 [40] are heavily upregulated in many invasive mammary carcinomas [41]. ERBB2/3 has also been linked to ovarian, bladder, stomach, salivary and lung carcinomas [42]. Increased ERBB2/3 alters multiple signaling pathways, including kinase signaling, and, thus, impairs normal cellular control mechanisms, giving rise to malignant tumor cell transformation [42]. This implies that the reduction of miR-125b may play a significant role in the development of these tumors [42].

In the following paragraphs, the oncogenic and tumor suppressive potential of miR-125b in the different tumor entities is summarized, with special focus on signaling cascades targeted by miR-125b and their implications in tumor formation.

### The oncogenic potential of miR-125b

**Figure 1:**
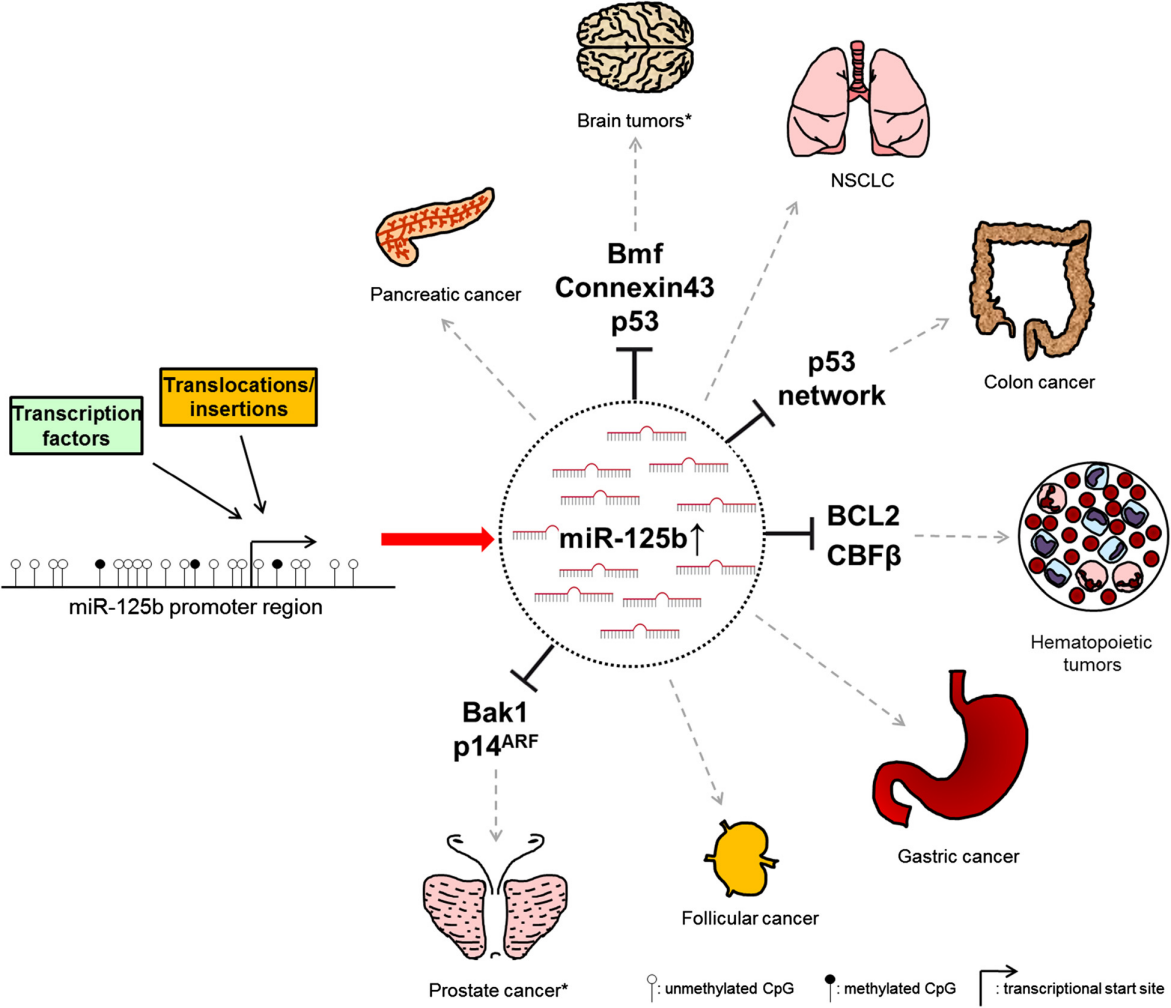
**“The bad guy”: miR-125b associated with oncogenic signaling in cancer.** t (2;11) (p21, q23) translocations, miR-125b-1 insertions and androgen signaling have been shown to cause an upregulation of miR-125b. This blocks the translation of target mRNAs which might promote the formation of the indicated tumor types. See also Table 1. *Upregulation of miR-125b in brain tumors and prostate cancer is controversially discussed, see main text.

In several tumor types elevated miR-125b levels have been detected. MiR-125b appears to downregulate anti-apoptotic proteins, resulting in reduced apoptosis and enhanced cellular proliferation, thereby promoting tumor growth (see Figure 1 and Table 1). The following miR-125b targets have been identified in different cancers:

**Table 1:** miR-125b associated with oncogenic signaling in cancer

**Tumor type**	**Direction of misregulation**	**miR-125b targets**	**Comments**	**Ref.**
Hematopoetic tumors	Up	BCL2	Regulator of apoptosis	[26]
	Up	CBFβ	Counteracts myeloid cell differentiation	[27]
	Up	Trp53inp1	Regulator of apoptosis	[43]
	Up	NA	NA	[25]
Colon cancer	Up in colon cancer with poor prognosis	p53	Prognostic indicator for colorectal cancer	[38]
Non-small-cell lung cancer (NSCLC)	Up in serum from patients with poor prognosis	NA	Prognostic serum marker for NSCLC	[44]
Follicular cancer	Up	NA	NA	[29]
Gastric tumors	Up	NA	Pro-proliferative, anti-apoptotic	[28]
Pancreatic cancer	Up	NA	NA	[30]

NA: not analyzed.

### Hematopoietic tumors

Chromosomal translocations cause a severe upregulation of up to 90-fold of miR-125b in myelodysplastic syndrome (MDS), acute myeloid leukemia (AML) and B-cell acute lymphoid leukemia (B-ALL). In these cases, miR-125b overexpression is the only consistent abnormality found, suggesting that it is the main oncogenic event [34,35,45]. One study very elegantly demonstrated that miR-125b overexpression is indeed the sole oncogenic event required for the development of leukemias: Bousquet *et al.* generated retroviral vectors to overexpress miR-125b and transplanted mice with fetal liver cells that were infected with the virus. 16 weeks post transplantation the animals showed a significant increase in white blood count associated with macrocytic anemia, suggesting pro-proliferative properties of miR-125b. Half of the mice transplanted with miR-125b overexpressing fetal liver cells succumbed to hematopoietic malignancies with different phenotypes, including malignant B-ALL, T-cell AML or myeloproliferative neoplasms within 12 to 29 weeks post transplantation [25]. The authors additionally showed that miR-125b overexpression can also be a secondary event that potentiates the effect of other oncogenes *in vivo,* such as BCR-ABL-induced leukemia [25]. Two additional studies confirmed the oncogenic potential of miR-125b in the hematopoietic system [43,46]: Overexpression of miR-125b in hematopoietic stem cells caused a dose-dependent myeloproliferative disorder that progressed to lethal myeloid leukemia in mice [46]. Further, transgenic mice mimicking the t (11;14) (q24; q32) translocation found in human B-cell precursor acute lymphoblastic leukemia that causes overexpression of miR-125b, developed lethal B-cell malignancies with clonal proliferation [43]. Subsequent *in vitro* experiments identified B-cell lymphoma-2 (BCL2), core-binding factor subunit β (CBFβ) and transformation related protein 53 inducible nuclear protein 1 (Trp53inp1) as direct miR-125b targets in myeloid cells, thereby counteracting pro-apoptotic pathways and myeloid cell differentiation [26,27,43].

### Colon cancer

MiR-125b also plays a crucial role in colorectal cancer. High levels of miR-125b expression in colorectal tumors are associated with reduced survival rates, most likely by directly targeting the p53 network and its downstream signaling molecules, such as p21, and thereby reducing pro-apoptotic stimuli [38].

### Non-small-cell lung cancer (NSCLC)

A study by Yuxia *et al.* demonstrated that miR-125b levels are elevated in serum of NSCLC patients by 10-fold [44]. It has recently been demonstrated that miRNAs are secreted within exosomes and may act on other cells taking up the exosomes. Transiting miRNAs can be detected in body fluids and may serve as a biomarker for NSCLC and other diseases [47]. Indeed, increasing miR-125b levels in serum are correlated to more malignant NSCLC stages as well as poor survival [44]. A second study confirmed the correlation of miR-125b serum levels and poor survival rates as well as poor response to cis-platin chemotherapy, suggesting that serum levels of miR-125b might be a useful prognostic and therapeutic marker for NSCLC [48]. No such correlation has been found for miR-125b expression in primary tumor tissue so far.

### Others

In follicular cancers, gastric tumors and pancreatic cancer miR-125b expression has been found to be increased. In gastric tumors miR-125b displays oncogenic, pro-proliferative characteristics [28-30].

### The tumor suppressive potential of microRNA 125b

**Figure 2:**
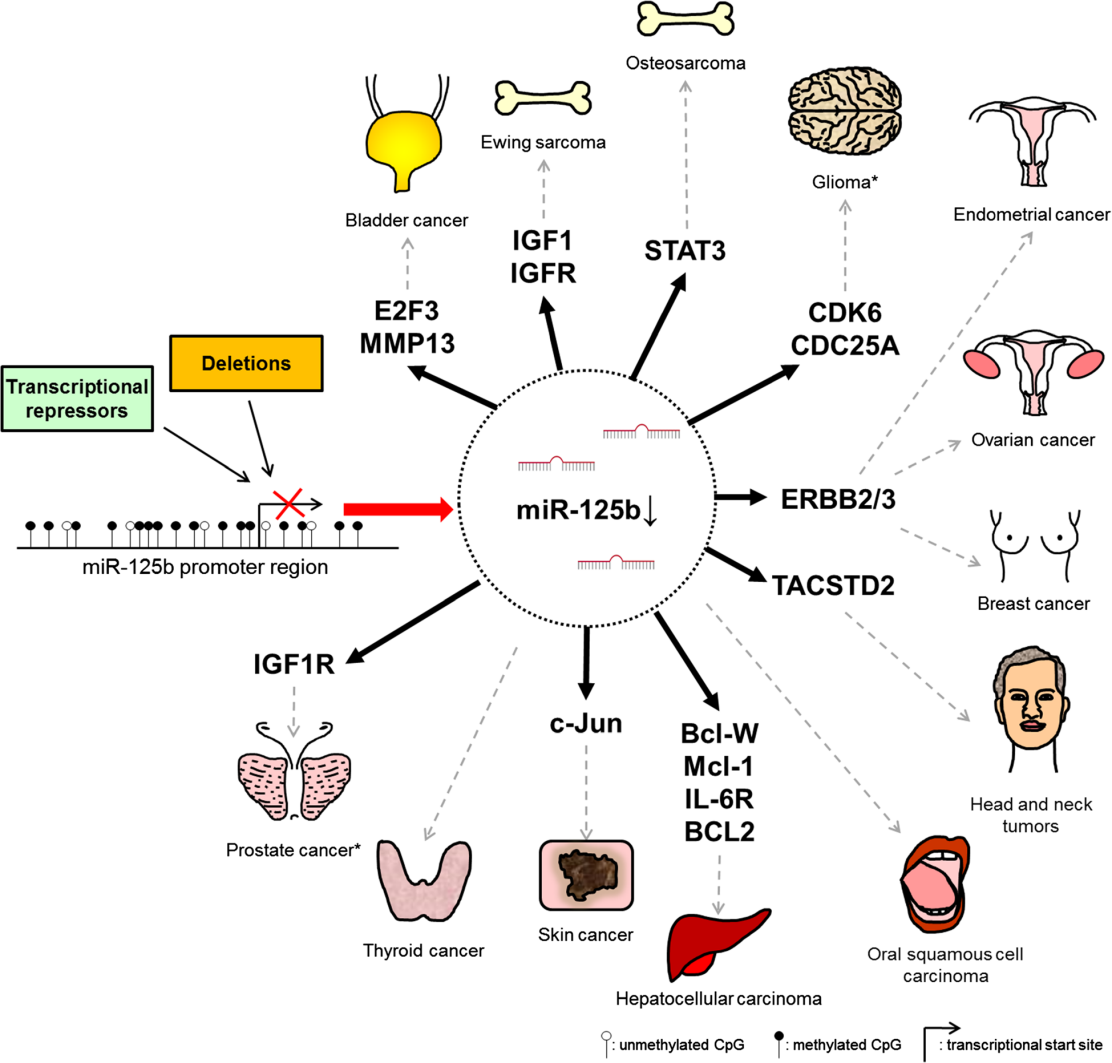
**“The good guy”: miR-125b associated with tumor suppressive signaling in cancer.** MiR-125b deletions, DNA hypermethylation and androgen signaling have been described to reduce miR-125b expression. This in turn causes the upregulation of multiple target mRNAs which might promote the formation of the indicated tumor types. Only the best characterized miR-125b target mRNAs are listed. For a complete list, see Tables 2 and 3. *Downregulation of miR-125b in gliomas and prostate cancer is controversially discussed, see main text.

Tumor suppressive potential of miR-125b has been described for multiple tumor types, in which miR-125b levels are decreased. Next to inhibition of cell growth and pro-apoptotic stimuli, miR-125b targets numerous proteins that alter kinase signaling, cellular migration and invasion as well as angiogenesis (see Figure 2 and Table 2).

**Table 2:** miR-125b associated with tumor suppressive signaling in cancer

**Tumor entity**	**Direction of deregulation**	**miR-125b targets**	**Comments**	**Ref.**
Bladder cancer	Down	E2F3	Regulator of colony formation, cell division	[49]
	Down	MMP13	Regulator of cell migration and invasion	[50]
	Down	NA	NA	[51]
Breast cancer	Down	EPO, EPOR	Regulator of differentiation and survival of erythroid cells	[52]
	Down	ETS1	Regulator of cell proliferation, clonogenicity and cell cycle progression	[24]
	Down	ENPEP, CK2-α, CCNJ, MEGF9	Cell proliferation and anchorage-independent growth	[53]
	Down	ERBB2/ERBB3	Regulator of migration and invasion	[40]
	Down	MUC1	Regulator of proliferation and apoptosis induction	[54]
Endometrial cancer	Down	ERBB2	Regulator of cell invasion	[20]
Ewing sarcoma	Down	IGF1, IGFR, mTOR, RSK1	Growth inhibitory properties	[55]
	Down	PI3K/Akt/mTOR	Regulator of proliferation, migration and invasion	[56]
Head and neck tumors	Down	TACSTD2 (TROP2)	Causes mitogen-activated protein kinase pathway dysfunction	[17]
Hepatocellular carcinoma (HCC)	Down	Bcl-W, Mcl-1, IL-6R	Regulator of apoptosis	[57,58]
	Down	BCL2	Regulator of apoptosis	[59]
	Down	PIGF	Regulator of invasion/angiogenesis	[60]
	Down	LIN28B	Regulator of cell migration and invasion	[61]
	Down		Suppresses the cell growth via Akt phosphorylation	[62]
	Higher in HCCs with good survival			
Oral squamous cell carcinoma	Down	NA	Regulator of proliferation	[21]
Osteosarcoma	Down	STAT3	Regulator of proliferation and migration	[22]
Ovarian cancer	Down	BCL3	Regulator of proliferation and clonal formation	[63]
	Down	HER2/HER3	Regulator of proliferation and angiogenesis	[64]
Skin cancer	Down	c-Jun	Regulator of proliferation	[19]
Thyroid cancer	Down	NA	NA	[29]

NA: not analyzed.

### Bladder cancer

MiR-125b is significantly downregulated in bladder cancer tissue by about 50% as well as in bladder cancer cell lines [49-51]. Overexpression of this miRNA in bladder cancer cell lines suppresses the potential to form colonies and to develop tumors in nude mice [49-51]. mRNA expression of the cell cycle transition molecule E2F transcription factor 3 (E2F3) is inversely correlated to miR-125b expression in cancerous tissues and cell lines, implying a miR-125b dependent regulation of E2F3 [49]. Cell invasion and migration is also regulated by miR-125b in bladder cancer cell lines. Matrix metalloproteinase 13 (MMP13) most likely mediates this effect, which has been demonstrated to be directly targeted by miR-125b [50].

### Breast cancer

MiR-125b is heavily downregulated in malignant breast tumors [24,40,52]. Reduced expression levels of miR-125b are most likely due to enhanced DNA methylation in the promoter regions, as demonstrated in breast cancer cell lines as well as mammary carcinoma tissue [24]. Further, deletions of the miR-125b loci have been described for human breast cancer tissues [16]. Computational approaches were used to identify potential miR-125b targets. Two of the first miR-125b targets that were identified and verified are ERBB 2 and 3 [40]. Activating ERBB2/3 expression and signaling by miR-125b downregulation induces cellular proliferation via the Ras-mitogen activated protein kinase (MAPK) signaling pathway and inhibits programmed cell death via the mammalian target of rapamycin (mTOR) pathway. Strikingly, these effects are more prominent in transformed breast cancer cell lines compared to non-transformed human mammary epithelial cells [40]. Further, downregulation of miR-125b in metastatic breast cancer induces the expression of erythropoietin (EPO) and its receptor EPOR also via the ERBB2/Her2 pathway, promoting cellular survival [52]. Also, miR-125b downregulation induces the expression of the proto-oncogene v-ets avian erythroblastosis virus E26 oncogene homolog 1 (ETS1), involved in cell cycle transition, cell growth and proliferation. Interestingly, ETS1 is overexpressed in invasive breast cancer samples [24]. Glutamyl aminopeptidase (ENPEP), casein kinase II subunit α (CK2-α), cyclin J (CCNJ), and multiple epidermal growth factor-like-domains 9 (MEGF9) are additional miR-125b targets that are upregulated in human breast cancer samples and may contribute to tumor progression [53].

### Endometrial cancer

One study showed that miR-125b is downregulated by approximately 30% in endometrioid endometrial cancer [20]. Downregulation of miR-125b in human endometrial cancer cell lines increased cell invasiveness which could be rescued by miR-125b overexpression. This effect is most likely mediated by the miR-125b target ERBB2 [20].

### Ewing sarcoma

In this pediatric malignancy, a proto-oncogenic fusion protein named EWS/Fli1 inhibits expression of a group of miRNAs, including miR-125b [55]. Interestingly, miR-125b has many predicted targets in the IGF-signaling pathway which is a key driver of Ewing carcinogenesis. One of these targets is ribosomal protein S6 kinase A1 (RSK1) which is directly downregulated by miR-125b in Ewing sarcoma cell lines, resulting in reduced cell growth and proliferation [55]. A second study identified the PI3K/Akt/mTOR pathway to be regulated by miR-125b in Ewing sarcoma cell lines, confirming its tumor suppressive potential [56].

### Head and neck tumors

In head and neck squamous cell carcinoma, DNA hypermethylation in the promoter region of miR-125b reduces the expression of this miRNA by about 100-fold [17]. Tumor-associated calcium signal transducer 2 (TACSTD2), a cell-surface glycoprotein, has been reported to be overexpressed in most epithelial tumors and was validated as miR-125b target in head and neck cancer. Disinhibition of TACSTD2 aberrantly induces MAPK signaling promoting tumor growth and proliferation [17].

### Hepatocellular carcinoma

Several studies have identified reduced miR-125b expression in liver cancer [57]. Multiple miR-125b targets were found to be upregulated in hepatocellular carcinoma, including BCL2, BCL2 like 2 (Bcl-W), myeloid cell leukemia sequence 1 (Mcl-1), interleukin-6 receptor (IL6R), lin-28 homolog B (LIN28B) and placenta growth factor (PIGF). This in turn inhibits the induction of apoptosis, promotes tumor angiogenesis, cell migration and invasion of cell lines and, thus, promotes carcinogenesis [57-62]. Strikingly, higher expression of miR-125b in hepatocellular carcinoma correlates with better survival rates, most likely by reducing Akt phosphorylation levels and subsequent inhibitory effects on cell growth and proliferation [62]. This further confirms the tumor suppressive nature of miR-125b in this tumor entity.

### Osteosarcoma

In osteosarcomas, miR-125b is reduced about 2.3-fold compared to adjacent non-cancerous tissue [22]. Accordingly, overexpression of miR-125b suppresses human osteosarcoma cell proliferation and migration *in vitro* as well as tumor growth in nude mice [22]. The signal transducer and activator of transcription 3 (STAT3) is directly targeted by miR-125b in osteosarcoma cell lines and, interestingly, alters the expression of miR-125b in a regulatory feedback loop, suggesting that tightly controlled levels of STAT3 are crucial for normal tissue homeostasis [22].

### Ovarian cancer

Ovarian cancer shows reduced miR-125b levels by approximately 75% compared to normal tissue [63,64]. Thus, overexpression of miR-125b in ovarian cancer cell lines does not only induce cell cycle arrest and inhibit proliferation, but also reduces tumor growth in a xenograft model [63]. These growth inhibitory effects of miR-125b are mediated by the miR-125b target B-cell CLL/lymphoma 3 (BCL3). Next to BCL3, ERBB2/3 (Her2/Her3) are also downregulated upon miR-125b overexpression in human ovarian cancer cell lines, further demonstrating that this pathway is involved in the development of multiple tumor types in which miR-125b is tumor suppressive. ERBB2/3 regulates angiogenesis in the chicken chorioallantoic membrane assay via the Akt/p70S6K1/HIF-1α/VEGF pathway [64]. Strikingly, ERBB2/3 has been shown to be upregulated in ovarian cancer tissues, suggesting enhanced angiogenenesis upon miR-125b downregulation in this tumor entity [64].

### Skin cancer

The expression of miR-125b is significantly reduced in malignant melanoma cell lines and tissue samples compared to melanocytes [19]. Forced expression of miR-125b suppresses proliferation and migration in melanocytes, confirming a tumor suppressive function of miR-125b. Overexpression of miR-125b is accompanied by reduced c-Jun protein expression in melanoma cells. C-Jun controls a wide set of substrates that are relevant for cell cycle, proliferation and differentiation [19], implying that miR-125b deregulation promotes c-Jun signaling and, therefore, carcinogenesis in malignant melanoma.

### Dual role of miR-125b

While in the above listed tumor types, miR-125b has a distinct oncogenic or tumor suppressive function, the role of miR-125b is controversially discussed in several other tumor entities. In those tumor types, miR-125b expression is elevated in tumor tissue compared to normal tissue and miR-125b targets that suggest oncogenic potential have been identified. However, other studies describe a downregulation of miR-125b and identify pathways that are regulated by miR-125b which rather imply tumor suppressive functions in those tumors (see Table 3).

**Table 3:** miR-125b associated with oncogenic and tumor suppressive signaling in cancer

**Tumor type**	**Direction of misregulation**	**miR-125b targets**	**Comments**	**Ref.**
Brain tumors	Up in ATRA differentiated glioblastoma cell lines	Bmf	Regulator of apoptosis	[31]
	Up	Connexin43	Anti-apoptotic, pro-proliferative	[65]
	Up in neuroblastoma cells	p53	Regulator of apoptosis	[32]
	Down	MAZ	Regulator of angiogenesis	[18]
	Down in glioma stem cells	CDK6 and CDC25A	Regulator of stem cell proliferation	[66]
Prostate cancer	Up in androgen-independent prostate cancer	Bak1	Pro-proliferative	[67]
	Up	p14^ARF^	Pro-proliferative, anti-apoptotic	[68]
	Down in androgen-treated prostate cancer cells	IGF1R	Anti-proliferative	[69]
	Down	NA	Translational control	[70]

NA: not analyzed.

### Brain tumors

MiR-125b is most abundantly expressed in the brain, where it is involved in neurogenesis and neural development by repressing multiple targets [71]. Given its pro-proliferative nature, two studies analyzed the effects of miR-125b overexpression in human glioma cell lines [31,65]. By targeting Connexin 43, an astrocytic gap junction protein that is commonly downregulated in astrocytomas [72], and Bmf (Bcl-modifying factor), miR-125b protects glioma cells from apoptosis *in vitro* and promotes human glioma cell proliferation. These findings were confirmed *in vivo*, in which transplanted miR-125b overexpressing glioma cell lines displayed enhanced cell growth and tumor size [65]. However, the current literature controversially discusses whether miR-125b is solely oncogenic in brain tumors: two studies describe a rather tumor suppressive function of miR-125b in brain derived cells [18,66]. Smits *et al.* show that miR-125b is critical for the regulation of glioma stem cell proliferation as it directly targets the cell cycle regulators cyclin dependent kinase 6 (CDK6) and cell division cycle 25 homolog A (CDC25A). MiR-125b expression is low in CD133-positive stem cells, leading to the upregulation of these two cell cycle regulators promoting growth and proliferation [66]. In line with these findings, another study demonstrates that miR-125b is downregulated in glioblastoma-associated endothelial cells, resulting in increased expression of its target, myc-associated zinc finger protein (MAZ), a transcription factor that regulates vascular endothelial growth factor (VEGF) [18]. In brain blood vessels of glioma patients, MAZ protein expression is also elevated, leading to enhanced VEGF signaling that supposedly shuts down miR-125b expression in a feedback loop [18], suggesting that miR-125b acts as a tumor suppressor.

While the oncogenic potential of miR-125b was analyzed in glioma cell lines, the tumor suppressive function of miR-125b was investigated in glioma stem cells and glioblastoma-associated endothelial cells. The usage of different cell types may account for the opposite results and future analyses of human glioma tissues will be needed to elucidate under which conditions miR-125b acts rather oncogenic or tumor suppressive in brain tumors.

### Prostate cancer

The role of miR-125b in prostate cancer is also controversially discussed. Several studies report an upregulation of miR-125b in malignant prostate cancer cell lines as well as clinical tissues of prostate cancer [67,70]. Shi *et al.* report that miR-125b expression is induced by androgen signaling, as androgens can bind to the promoter region of miR-125b. This in turn downregulates the anti-apoptotic proteins BCL2 homologous antagonist/killer 1 (Bak1) and p14^ARF^ and, thus, promotes proliferation of androgen independent prostate cancer [67,68]. The upregulation of miR-125b by androgens has been further demonstrated in a more recent study investigating androgen-mediated ovarian follicular development, leading to the repression of pro-apoptotic signaling events [73]. In contrast, another study describes an inhibitory effect of androgen treatment on the expression of a miRNA cluster, containing miR-125b, in prostate cancer cell lines [69]. This results in the upregulation of multiple miR-125b target genes, including insulin-like growth factor 1 receptor (IGF1R). Accordingly, the authors conclude that androgen-repression of miR-125b promotes androgen-dependent growth of prostate cancer cell lines through the de-repression of IGF1R. Future studies will be needed to fully elucidate the implications of androgen-signaling on miR-125b expression and the precise role of miR-125b deregulation in prostate carcinogenesis.

It is important to highlight that most of the studies described in the paragraphs above measured miR-125b expression levels in heterogeneous tissue extracts and not purified cancer cells. Thus, the up- or downregulation of miR-125b could be a consequence of changes in cellular composition of the tumors compared to normal tissue rather than a specific miR-125b activating or repressing event. Thus, tissue heterogeneity might account for the opposing results described for brain tumors and prostate cancer.

## Conclusion

The six classical hallmarks of cancer are escape from growth suppression, maintenance of proliferation, resistance to cell death, induction of angiogenesis, activation of invasion and metastasis and replicative immortality [74]. MiR-125b modulates several of these pathways through multiple target genes, resulting in either oncogenic or tumor suppressive modes of action, which contribute to or inhibit carcinogenesis, respectively. Several hypotheses, why miR-125b exerts oncogenic or tumor suppressive potential, depending on the tissues analyzed, might explain the opposing roles of miR-125b in different cancers:

### Same, same, but different – the same miR-125b targets can have diverse effects in individual tissues

Multiple target genes have been described for miR-125b, which have been shown to be more or less relevant in tumor initiation and progression in different tissue types. For example, many solid tumors have mutations in the tumor suppressor gene p53. However, while the vast majority of colorectal tumors and NSCLC carry p53 mutations (see http://p53.free.fr/index.html), the absolute frequency of p53 mutations in breast cancer is significantly lower [75]. Consequently, overexpression of miR-125b that further reduces p53 expression and activity in colon and lung cancer tissue promotes carcinogenesis by blocking the apoptotic machinery [76]. Thus, miR-125b acts as classical oncogene in those tumors. This is further supported by the finding that miR-125b expression levels are correlated with reduced survival rates in these entities [38,44]. In contrast, the observed downregulation of miR-125b in mammary tumors and other cancer tissues might actually inhibit tumor growth through increased p53 expression. However, the increase in expression of other miR-125b targets, such as the EGF receptor family members ERBB2/3 might be overall more relevant in breast cancer tissue, as overexpression of this gene is one of the most prominent oncogenic drivers in mammary carcinoma. ERBB2/3 has been shown to be overexpressed in breast, ovarian, bladder, stomach, and salivary carcinomas, thereby impairing normal cellular control mechanisms and giving rise to malignant tumor cell transformation [42]. Interestingly, miR-125b is heavily downregulated in several of these ERBB2/3-driven tumor entities [12], allowing for further ERBB2/3 upregulation [40,64], thereby promoting malignant transformation.

Hence, due to tumor specific activation/inactivation of miR-125b regulated signaling pathways both upregulation and downregulation of miR-125b may promote carcinogenesis in different tumors. For example, miR-125b induction may promote oncogenic pathways in some tumor types predominantly via the inhibition of the p53 axis, e.g., in the case of colon carcinoma and NSCLC [31,32,59]. On the contrary, downregulation of miR-125b in other tumor types induces oncogenic pathways, e.g., ERBB2/3 signaling in mammary carcinoma [40,41]. This suggests that the combination of balanced levels of miR-125b as well the expression of its target genes is crucial for proper physiological function.

### Overexpression or downregulation of miR-125b induces tissue specific oncogenic or tumor suppressive functions, respectively

MiR-125b targets both oncogenes and tumor suppressor genes. As summarized above, the overexpression of miR-125b in tissues, e.g., the hematopoietic system [34,35,45] or colorectal tumors [38], is an oncogenic event, as anti-apoptotic proteins are downregulated. In contrast, downregulation of miR-125b is associated with loss of tumor suppressive modes of action, e.g., in mammary [24,40,52] or ovary tissue [22], since pro-proliferative proteins are upregulated. Therefore, one can hypothesize that miR-125b upregulation dictates oncogenic characteristics, while downregulation of miR-125b corresponds to the loss of tumor suppressive functions by this miRNA. This is further supported by the fact that overexpression of miR-125b has never been correlated to tumor suppressive functions and that loss of miR-125 has never been shown to activate tumor suppressive pathways *in vivo* so far.

But what determines upregulation or downregulation of miR-125b in different tumor types?

Not much is known about the transcriptional regulation of miR-125b. Expression profiles of cancer samples compared to normal tissue indicate that global miRNA downregulation is a very common event during carcinogenesis [77]. The downregulation of miR-125b is observed in multiple tumors, but the molecular mechanisms have been elucidated only for a few cancer types so far. It is believed that genotoxic events repress miR-125b expression [32]. In mammary, cervical and ovarian tumors, deletions at fragile sites have been described that cause the deletion of the miR-125b-1 gene [16], which implies a loss-of-function mechanism, as reduced miR-125b levels allow for oncogene activation in those entities. Another mechanism that shuts down miR-125b expression is DNA hypermethylation that has been described for head and neck tumors and invasive breast cancer [23,24]. Promoter DNA methylation is known to be a rather specific event during carcinogenesis, since only selected genes are hypermethylated in a tumor cell [78]. This suggests that tissue specific silencing of the tumor suppressor miR-125b in head and neck tumors and breast cancer by DNA methylation confers a selective advantage to the cells that does not occur in other tumor entities. It is very likely that tissue specific transcriptional regulators, such as enhancers/repressors and transcription factors control miR-125b expression [79] that can have particular consequences: By now it is very well appreciated that miRNAs function by repressing target mRNAs that should not be expressed (reviewed in [77]). Loss of miR-125b expression consequently enables target gene expression even in the absence of signaling events. This promotes carcinogenesis, e.g. by ERBB2/3 upregulation, irrespective of the molecular mechanisms leading to miR-125b loss. Further, miRNAs and their targets are very often co-activated or co-repressed by the same incoming signal. This guarantees that unwanted stochastic signaling events do not cause activation or repression of target gene expression and that signaling fluctuations are efficiently buffered (reviewed in [77]). Hence, miRNAs balance and buffer signaling events, crucial for proper tissue homeostasis. In the case of miR-125b downregulation, this causes a loss of tumor suppressive control mechanisms leading to aberrant activation of multiple oncogenes in a tissue specific manner (see above).

The upregulation of miR-125b might regulate different signaling pathways in varying cell types. For instance, miR-125b could repress a set of tumor suppressors prominently expressed in one type of cells, thereby serving as an oncomiR. In a different cell type or when using cancer cell lines that have different transcriptional programs with specific oncogenes prominently expressed, miR-125b overexpression might repress the expression of these oncogenes and thereby serve as a tumor suppressor. Hence, the upregulation of miR-125b is a specific oncogenic event that has been described for a few tumor entities only. Mutations, deletions, chromosomal translocations, copy number variations or any other genetic alteration that changes expression levels are believed to be stochastic events in sporadic tumor formation that equip the cell with tumor initiating/promoting advantages compared to non-transformed cells [80]. Chromosomal translocations are a classical hallmark of hematopoietic tumors, and occur at a much higher frequency than in other malignancies [81]. In MDS, AML and B-ALL, chromosomal translocations at the miR-125b locus cause an almost 100-fold induction of miR-125b expression, which is the major oncogenic event in these cases [34,35,45]. This provides the cells with a growth advantage since anti-apoptotic pathways are downregulated. Such miR-125b-inducing translocations have not been described for solid tumor tissues, most likely because chromosomal translocations occur much less frequently [81]. As a result, upregulation of miR-125b expression by chromosomal translocations is a very rare event that is restricted to hematopoietic tumors, ultimately leading to tissue specific oncogenic miR-125b signaling. In contrast, in Ewing sarcoma, a chromosomal translocation involving a transcription factor inhibits the expression of the miRNA-cluster in which miR-125b is located, leading to repression of miR-125b expression [55]. This in turn de-represses oncogenic signaling leading to tumorigenesis.

MiR-125b is upregulated in a few other tumor types, such as prostate cancer [67,70]. In prostate cancer, the induction of miR-125b has been linked to elevated androgen signaling [67], which is a hallmark of prostate carcinogenesis and promotes androgen-dependent proliferation in early stage prostate cancer [82]. Androgen-dependent upregulation of miR-125b is most likely restricted to prostate tissue only. The causes for miR-125b upregulation in colorectal cancer and brain tumors are unknown. Nevertheless, since miR-125b targets very important tumor suppressor genes, such as the p53 network, overexpression of miR-125b is a clear oncogenic event that promotes carcinogenesis in the tissues in which upregulation occurs.

### Limitations and future directions

Current data on the pathomechanism of miR-125b in carcinogenesis were mostly generated in immortalized cell lines and were only partially verified *in vivo*. In some cases xenograft models were used to demonstrate the tumor suppressive potential of miR-125b *in vivo*: it was shown that miR-125b overexpression inhibits tumor formation by inducing apoptotic pathways using bladder cancer cell lines [49-51], osteosarcoma cell lines [22] and ovarian cancer cell lines [63]. It is noteworthy that those studies generally show that altered miR-125b expression affects cell growth *in vitro* and in xenograft models rather than measuring *de novo* tumorigenesis. Only a few studies clearly demonstrated real oncogenic potential of miR-125b *in vivo*: The transplantation of fetal liver cells and hematopoietic stem cells overexpressing miR-125b, as well as a transgenic mouse model mimicking the t (11;14) (q24; q32) translocation induced leukemia in mice [25,43,46], proving that miR-125b is a classical oncogene in the hematopoietic system.

Nevertheless, these models represent very valuable tools to study principle functions of miRNA signaling and provide further insights into the role of miR-125b targets in carcinogenesis. However, they do not reflect the full picture of tumorigenesis as foreign cells with dramatically forced expression of miR-125b (in the leukemia mouse model, retrovirus induced miR-125b expression was up to 800-fold above normal levels) were administered into immune compromised animals [83]. Therefore, for the future study of miR-125b function, transgenic miR-125b animal models will be needed which allow for specific overexpression/knockdown in individual cell types and tumor types that reflect better the actual situation in human cancer patients. Especially miR-125b knockout models in specific tissue types will be needed to address the question whether miR-125b truly is a tumor suppressor and absolutely relevant for proper tissue homeostasis. Such studies will reveal in more detail how aberrant miR-125b expression, as it is observed in human malignancies, contributes to the malignant transformation of tumors *in vivo*.

In summary, miR-125b is a “double-edged” miRNA that has multiple targets which control pro-proliferative and pro-apoptotic signaling pathways in parallel and which has to be tightly regulated under physiological conditions. If this level of regulation is lost during carcinogenesis, oncogenic or tumor suppressive pathways are activated or blocked. Due to tissue specific signaling cascades, disturbing the miR-125b equilibrium can have opposing effects: if miR-125b is downregulated, the “good guy” with its tumor suppressive functions is lost, oncogenic pathways (e.g., ERBB2/3) are activated and pro-apoptotic cascades are repressed, resulting in malignant transformation. If the equilibrium is shifted towards miR-125b overexpression due to chromosomal translocations or transcriptional activation, miR-125b transforms into the “bad guy” and promotes oncogenic signaling by for example downregulating p53 and other apoptosis-inducing pathways.

In the future, the diverse roles of miRNAs in the pathogenesis of multiple diseases will be further established, and potential therapeutic strategies to target aberrantly expressed miRNAs might be successfully developed [84]. For this purpose, the in-depth understanding of miRNA signaling in different tissue types and diseases will be essential, in order to fully elucidate the precise function of individual miRNAs. This will provide the potential to not only stratify patients into different disease subgroups, but to also design individualized miRNA based novel therapies.

## Abbreviations

AML: Acute myeloid leukemia; ATRA: All-trans-retionic acid; Bak1: BCL homologous antagonist/killer 1; B-ALL: B-cell acute lymphoid leukemia; BCL2: B-cell lymphoma-2; BCL3: B-cell CLL/lymphoma 3; Bcl-W: BCL2 like 2; Bmf: Bcl-modifying factor; CK2-α: Casein kinase II subunit α; CBFβ: Core-binding factor subunit β; CCNJ: Cyclin J; CDC25a: Cell division cycle 25 homolog A; CDK6: Cyclin dependent kinase 6; E2F3: E2F transcription factor 3; ENPEP: glutamyl aminopeptidase; EPO: Erythropoietin; EPOR: Erythropoietin receptor; ERBB: v-erb-b2 avian erythroblastic leukemia viral oncogene homolog; ETS1: v-ets avian erythroblastosis virus E26 oncogene homolog 1; Her: Human epidermal growth factor receptor; HIF-1α: Hypoxia inducing factor 1 α; IGF1R: Insulin-like growth factor 1 receptor; IL6R: Interleukin-6 feceptor; LIN28B: Lin-28 homolog B.; MAPK: Mitogen activated protein kinase; MAZ: Myc-associated zinc finger protein; Mcl-1: Myeloid cell leukemia sequence 1; MDS: Myelodysplatic syndrome; MEGF9: Multiple epidermal growth factor-like domains 9; miRNA: microRNA; miR-125b: microRNA 125b; MMP13: Matrix metalloproteinase; mTOR: Mammalian target of rapamycin; p21: cyclin dependent kinase inhibitor 1; p53: tumor suppressor gene p53; PIGF: placenta growth factor; Pre-miRNA: Precursor microRNA; Pri-miRNA: Primary microRNA; Ref: Reference; RISC: RNA-induced silencing complex; RSK1: Ribosomal protein S6 kinase A1; STAT3: Signal transducer and activator of transcription 3; TACSTD2: Tumor-associated calcium signal transducer 2; Trp53inp1: Transformation related protein 53 inducible nuclear protein 1; VEGF: Vascular endothelial growth factor.

## Competing interests

The authors declare that they have no competing interest.

## Authors’ contributions

JBS and DE set up the outline of the review. JBS wrote the review with critical input from DE. Both authors read and approved the final manuscript.
